# Turning lances into shields: flower mantids stretch their raptorial forelegs to avert and deflect predator attack

**DOI:** 10.1098/rspb.2024.3081

**Published:** 2025-04-02

**Authors:** Yuanlang Li, Qinpeng Liu, Zhaoyang Chen, Le Liang, Zhixin Wang, Yuange Duan, Fan Song, Wanzhi Cai, Jin Ge, Hu Li, Li Tian

**Affiliations:** ^1^ State Key Laboratory of Agricultural and Forestry Biosecurity, MOA Key Lab of Pest Monitoring and Green Management, College of Plant Protection, China Agricultural University, Beijing 100193, People's Republic of China; ^2^ College of Biological Sciences, China Agricultural University, Beijing 100193, People's Republic of China; ^3^ State Key Laboratory of Integrated Management of Pest Insects and Rodents, Institute of Zoology, Chinese Academy of Sciences, Beijing 100101, People's Republic of China

**Keywords:** behavioural co-option, anti-predator defence, phylogenetic analysis, flower mantid

## Abstract

Evolutionary co-option, in which existing traits acquire novel adaptive functions, is a key strategy by which organisms adapt to new environmental challenges. Although such co-option has been widely documented at the genetic and morphological levels, its incidence at the behavioural level remains largely unknown. Mantids stretch their forelegs to capture prey; however, some flower mantids also perform foreleg stretches in the absence of prey. The current study tested whether this behaviour represents a novel function of the foreleg stretch, thus representing a case of behavioural co-option. Predator encounter behaviour assays revealed that foreleg stretching facilitates the escape of flower mantids from large predatory mantids by delaying predator approach or deflecting their attack towards less vulnerable body parts. Phylogenetic analysis suggested that the ancestral function of foreleg stretching involves prey capture, with the anti-predator function subsequently acquired in the flower mantid clade, coinciding with the diversification of large-sized mantids, the most likely invertebrate predators of flower mantids. This study provides a case of behavioural co-option, where a predator uses its predatory organ as a defensive implement to cope with its own predators. These findings further suggest that behavioural co-option may be common in nature, meriting more comprehensive studies.

## 1. Introduction

Evolutionary co-option, in which existing traits are modified to acquire novel adaptive functions, is common in nature [1,2]. As a significant interpretation of Darwin’s theory of evolution [1], co-option has been widely demonstrated at the molecular and morphological levels. Classical examples include butterflies, where genes that were initially involved in the context of eye pigmentation and appendage development are co-opted to regulate wing pattern formation [3–6], and birds, in which feathers that originally evolved for thermal regulation are co-opted for flight [2,7]. Studies on co-option have challenged the original definitions of homology and evolutionary novelty by demonstrating that new structures commonly evolve through modification of pre-existing structures rather than arising *de novo* [1].

In contrast to the extensive documentation of co-option at the genetic and morphological levels, behavioural co-option has received far less research attention. This may be largely due to the challenges associated with manipulative experiments for empirically testing behavioural function, as well as the difficulties of conducting cross-species behavioural analyses within a phylogenetic framework to assess the functional history of a behavioural trait [8]. To date, documentation of behavioural co-option has been limited to vertebrates and restricted to social communication behaviours involving *ritualized signals* [9–12]. For example, previous studies demonstrate that the courtship signals of bowerbirds are co-opted from aggressive displays, and the head-down threat behaviour of stickleback fish is co-opted from digging or biting behaviours [9,11,12]. Despite these classical cases, several limitations remain in our understanding of behavioural co-option. First, it is unclear whether co-option exists in other behavioural contexts beyond ritualized intraspecific communication. Second, previous studies have mainly focused on vertebrates; therefore, it remains unknown whether behavioural co-option occurs across a wider taxonomic range, including invertebrate taxa. Moreover, most previous studies on behavioural co-option lack analyses of the evolutionary history of the behavioural traits, thereby limiting our understanding of how and when the behaviour of interest acquired novel functions, which is crucial for further understanding the ecological forces driving behavioural co-option [1,12]. Consequently, it remains unknown whether co-option at the behavioural level is as common as that occurring at the molecular and morphological levels.

Mantids are well characterized by their elongated, spiny raptorial forelegs and their highly efficient hunting behaviour, which includes rapidly stretching their forelegs to capture moving prey [13]. However, a group of small-sized mantids in the family Hymenopodidae, known as flower mantids [14], display conspicuous markings on their femurs [15] and stretch their raptorial forelegs to expose these markings even in the absence of prey [16,17]. This suggests that the foreleg-stretching behaviour of flower mantids may have acquired a novel function in addition to its original predatory purpose, indicating a likely case of behavioural co-option. Mantids possess a relatively robust phylogeny [18–20], and many species, including flower mantids, are easy to rear in laboratory conditions. Some mantid species have already been used as models for behavioural and developmental genetic studies [21–24]. These characteristics offer a suitable model for manipulative laboratory behavioural experiments and phylogenetic analyses to examine the evolutionary history of behavioural traits, which are important for empirically studying behavioural co-option.

Many animals with conspicuous colour markings also exhibit unique marking display behaviours, which play roles in various ecological contexts such as intra- and interspecific communication. For example, the males of the jumping spider *Habronattus pyrrithrix* display colourful ornaments to females during courtship for sexual communication [25]. Although it has been speculated that flower mantids stretch their colour-marked forelegs to avoid conspecific cannibalism [16], this hypothesis fails to explain why some of the species continue to display foreleg stretching in the absence of conspecific encounters [16,17]. Alternatively, marking displays can also function as a defensive strategy. Indeed, many insects and amphibians use conspicuous markings to intimidate predators [26–28]. For example, the rough-skinned newt *Taricha granulosa* reveals conspicuous patterns to intimidate predators when under attack [27]. Animals displaying colour markings also could function in deflecting predators’ attacks [29]. A notable example involves butterflies in the family Lycaenidae, which display head-like hindwings to direct a bird’s attack towards these less vulnerable body parts to facilitate escape [30–32]. Therefore, it is likely that the foreleg stretching of flower mantids can serve as a predator defence.

Aiming to examine whether the foreleg-stretching behaviour of flower mantids represents a case of behavioural co-option, we tested three hypotheses: (i) the foreleg-stretching behaviour in the flower mantid *Astyliasula basinigra* serves as a protective defence against predators, (ii) the novel function of foreleg-stretching behaviour is a derived function relative to its predatory function in evolutionary history, and (iii) the evolution of the anti-predator function of foreleg-stretching behaviour is driven by predation pressure. To test these hypotheses, we examined the biological function and evolutionary history of the foreleg-stretching behaviour through behaviour experiments and phylogenetic analysis. Our results suggest that behavioural co-option may be common in animals, providing a paradigm for testing behavioural co-option across taxa.

## 2. Material and methods

### (a) Experimental animals

#### (i) Rearing methods

All experimental mantids were collected from a local breeder in Yunnan and reared in laboratory conditions at China Agricultural University (40.00° N, 116.36° E), Beijing, China, following previously reported methods [23] with slight modifications. Specifically, mantids were housed individually in plastic rearing boxes and kept under controlled temperature of 26 ± 2°C, 40 ± 10% relative humidity, and a 16/8 h light/dark photoperiod (with light on between 6.00 and 22.00). Flower mantids were used in behavioural experiments at the fourth instar, when they were large enough for observation and the colour pattern of the forelegs became evident (see electronic supplementary material, table S1 for further rearing details).

#### (ii) Ethical considerations

According to Chinese regulations, this work did not require ethical approval from a human subject or animal welfare committee. The experiments were non-invasive, and the mantids were cared in laboratory by trained and competent staff, which included routine monitoring of welfare.

### (b) Predator encounter assay

#### (i) Experimental settings and mantid manipulations

To test whether foreleg stretching can help flower mantids evade their predators, we performed predator encounter assays using fourth instar nymphs of the flower mantid *A. basinigra* (*n* = 26) as the model and fourth instar nymphs of the large-sized mantid *Rhombodera longa* (*n* = 9) as the predator. We selected these two species as a predator–prey combination because they co-occur across southwest Yunnan and share similar habitats (electronic supplementary material, figure S1) [33–35].

To simulate a predator encounter scenario, we sequentially transferred one *A. basinigra* and one *R. longa* into a 20 cm × 20 cm arena. The arena consisted of six crossed sticks with graph paper at the bottom. To standardize the physiological condition and increase the activity level of the mantids, all candidate individuals, including the flower mantids and their predators, were starved for 2 days before trials. At the beginning of the trial, the flower mantid and the predator were given 5 min to acclimatize to the environment. To examine the effects of foreleg stretching on the survival of flower mantids during predator encounters, we compared survival outcomes among three behavioural groups: (i) the normal-stretching group, which included non-manipulated mantids that stretched their forelegs at the time of encountering a predator; (ii) the normal-non-stretching group, consisting of non-manipulated mantids that did not stretch their forelegs at the time of encountering a predator; and (iii) the manipulated non-stretching group, including mantids with physically damaged forelegs, who were unable to stretch their forelegs but could still walk freely (electronic supplementary material, figure S2). To disable the forelegs of the individuals, the foreleg coxae were slightly injured with a pointed tweezer (approx. 3 s). The normal groups contained 18 individuals, while the manipulated group contained eight individuals.

To record the behaviour of the two species and the survival outcome of the flower mantids, a mobile phone (FOA-AL00) was fixed above the arena. Experimental video recordings were used for subsequent behavioural analysis. If, in any trial, (i) the two mantids encountered each other within the acclimation period or (ii) one of them left the arena before the encounter, the recording was ended, and the trial was discarded.

#### (ii) Behavioural analysis

During video analysis, we documented two types of survival outcomes during predator encounters: ‘escape’ and ‘capture’. An ‘escape’ was defined as a successful evasion in which the flower mantid managed to distance itself from the predator by running or jumping [36]. A ‘capture’ event was identified when the mantid was firmly held by the predator’s forelegs for at least 3 s. The frequency of each survival outcome was compared among the behavioural groups.

Additionally, to examine the effects of foreleg-stretching behaviour on the predatory behaviour of *R. longa*, we recorded several aspects of predator behaviour, including whether an attack was initiated, the direction of head turning, the approaching speed of the predator and the direction of the attack. Specifically, an ‘attack’ was defined when the predator rapidly stretched its raptorial forelegs towards the flower mantid [21]. The direction of head turning of *R. longa* relative to the position of the flower mantid was measured to determine whether the predator’s attention was attracted to or deflected away from the flower mantid. Towards this end, we first drew a straight line connecting the centre of the flower mantid’s body and the end of the predator’s pronotum; the angle between the predator’s head direction and this straight line was then measured. We selected 20 pairs of images showing the flower mantids before and after stretching their forelegs to measure the deviation angle of the predator’s head. ImageJ (v. 1.54f, Wayne Rasband and contributors, National Institutes of Health, USA) software was used to measure the distance between the predator’s head and the flower mantid over time to assess whether the predator approached at a slower pace when the flower mantid stretched its forelegs. The direction of the attack by the predator was recorded based on the specific body part of the flower mantid the predator was targeting, including the forelegs or body trunk. In addition, if the predator initiated the attack but missed the flower mantid entirely, the direction was recorded as ‘missed’.

#### (iii) Statistical analysis on survival outcomes

Statistical tests were implemented using R Studio software, v. 2024.09.1+394, and R software, v. 4.2.2 (37). Both general linear mixed models (GLMMs) and Chi-square tests were used to assess the effects of the foreleg-stretching behaviour on escape rates. Chi-square tests were also employed to examine the effect of the foreleg-stretching behaviour on the attack rate of predators and the association between the mode of predator attack and the escape rate of flower mantids. Student’s *t*-tests were used to evaluate differences in the deviation angle of the predators’ heads among different behavioural groups. The change in relative distance between predators and flower mantids over time was analysed using linear regression.

### (c) Characterizing the types of foreleg-stretching behaviours

To examine the variation of foreleg-stretching behaviour across species, we compared the stretching behaviour among three flower mantid species: *A. basinigra*, *Pseudocreobotra wahlbergii* and *Acromantis hesione* (*n* = 4 for each species). Individual mantids were placed in a white background arena (30 cm × 20 cm × 15 cm) with graph paper at the bottom. The mantids were allowed 15 min to acclimatize to the arena before video recording, after which their behaviour was recorded from a back view and subsequently analysed on a computer.

We tracked the relative position of the apical point of the foreleg femurs to the body’s central axis and the central pronotum (electronic supplementary material, figure S3). For better visualization and quantification of the foreleg-stretching behaviour, we compiled a dataset comprising 1687 points from 38 videos, which included the central pronotum and the trajectory points of the femur apical point, recorded every five frames. ImageJ (v. 1.54f) was used to extract the trajectory points of the apical femur. The modes of foreleg stretching were characterized by the direction of the foreleg extension, represented by the average angles between the body’s central axis and the femur-pronotum line [38].

### (d) Analyses of the evolutionary history of foreleg-stretching behaviour

#### (i) Taxon sampling

To investigate the relationships and evolutionary history of foreleg-stretching behaviours, we conducted a series of evolutionary analyses. For the sampling setting, we curated a taxon set comprising 50 species across the main clades of Mantodea (electronic supplementary material, table S2) to achieve satisfactory calibrated coverage. The in-group dataset consisted of 28 species from Hymenopodidae, representing all tribal-level lineages within this family. We specifically focused on sampling lineages referred to as ‘boxer mantids’ and the ‘Oxypilinae + Acromantinae clade’ [15], comprising 14 species from 11 genera.

#### (ii) DNA extraction and mitogenome sequencing

We used mitochondrial genomes, comprising 13 protein-coding genes (PCGs), 2 rRNA genes and 3 nuclear genes (18S rRNA, 28S rRNA and histone 3), as molecular markers to reconstruct the phylogeny of Hymenopodidae. These gene markers were derived from whole-genome next-generation sequencing (NGS) of samples from 15 species conducted in this study, coupled with three publicly available transcriptomic NGS datasets from the Sequence Read Archive and GenBank for the remaining species (electronic supplementary material, table S2). Pre-processing of the data prior to the phylogenetic analyses, including quality control, assembly, annotation and alignment [39–42], was performed following protocols established by Liu *et al*. [20], resulting in a concatenated supermatrix comprising 17 057 nucleotide characters in Geneious [43] after removal of poorly aligned regions from each alignment using trimAl [44]. The concatenated supermatrix was partitioned based on each gene and the codon position of each PCG to determine the best-fit partitioning strategy and substitution models using PartitionFinder2 v. 2.1.1 [45] with the ‘models = all’ option (electronic supplementary material, table S3). The resulting partitioned nucleotide matrix was analysed using the maximum-likelihood method best-tree approach in IQ-TREE v. 1.6.12 [46].

#### (iii) Divergence time estimation and ancestral state reconstruction

The time of divergence between extant lineages was estimated using BEAST v. 1.10.4 (see electronic supplementary material, methods) [47]. To examine the evolutionary path of the foreleg-stretching behaviour, we performed ancestral-state reconstruction of this behaviour based on the phylogenetic tree. We first established extensive behavioural datasets by closely observing and recording the occurrences of foreleg-stretching behaviours throughout the life history of various mantodean species, including hymenopodids from different genera. For species that are difficult to obtain or rear, behavioural data were obtained from the literature and online videos (electronic supplementary material, table S4). Based on the assembled behavioural datasets, we prepared two matrices of character states for analyses in the R package ‘phytools’ [48], and the ultrametric topology derived from BEAST v. 1.10.4 was set as input, with lineages outside of Mantidea pruned out before analyses.

## 3. Results and discussion

### (a) Foreleg stretching is an anti-predator defence behaviour

**Figure 1.**
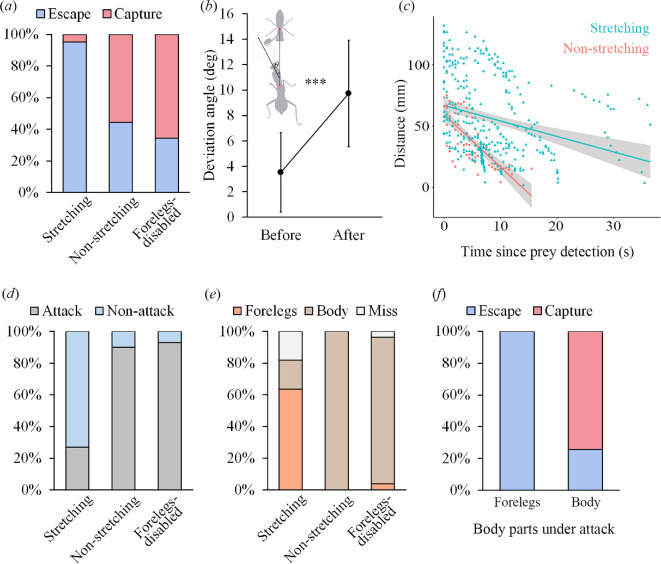
The effects of foreleg stretching in reducing predation risk. (*a*) Survivorship of the flower mantids during predator encounters, represented by the escape rate of the flower mantids in the three groups. (*b*) Foreleg stretching acts on predator deviation, indicated by the angle of the predator’s head deviation before and after the foreleg-stretching behaviour was performed. (*c*) Foreleg stretching delays the predator’s approach, as indicated by changes in the relative distance between the predators and the flower mantids following the predator’s detection of the prey. Slopes represent speeds (stretching: −1.26, *r*^2^ = 0.083, *p* < 0.01; no stretching: −4.30, *r*^2^ = 0.576, *p* < 0.01). (*d*) Foreleg stretching decreases the predators’ attack rate. (*e*) Effects of foreleg stretching on the attack direction of the predator mantids, represented by the attack towards the forelegs (Forelegs), the body trunk (Body) and those that completely missed the flower mantids (Miss). (*f*) Escape rate in the face of attacks towards the forelegs relative to attacks towards the body trunk.

Results showed that the foreleg-stretching behaviour enhanced the survival possibility of the flower mantids under predation risk. Specifically, foreleg stretching was observed in 41 trials, while it was absent in the remaining nine trials. We then compared survival outcomes between foreleg-stretching and non-stretching trials within the normal mantid group. The results showed that foreleg stretching during predator encounters was associated with significantly higher survival rates compared to those in non-stretching trials (stretching: 95% survival rate, non-stretching: 44% survival rate, *Z* = 3.13, *p* < 0.01, multinomial GLMMs; electronic supplementary material, table S5). Additionally, groups with a foreleg disability (which prevented foreleg stretching) exhibited a survival rate of only 35%, which was significantly lower than that of the normal foreleg-stretching group (*χ*^2^ = 26.93, *p* < 0.001; figure 1*a*). These findings suggest that foreleg stretching plays a critical role in enhancing escape success during predator encounters in flower mantids.

To investigate how foreleg stretching facilitates predator avoidance, we further examined the predator’s behavioural response to this action. Analysis of the predator’s head movement revealed that the deviation angle between the predator’s head and the position of the flower mantid significantly increased when the mantid stretched its forelegs (before stretching: 3.51 ± 3.13°, after stretching: 9.74 ± 4.19°; *t* = 5.32, *p* < 0.001; figure 1*b*). This suggests that foreleg stretching prompts the predator to shift its head away from the flower mantid’s position. Furthermore, we found that although predators continued to approach flower mantids regardless of whether the forelegs were stretched, they approached the prey at a significantly slower pace (figure 1*c*) and were much less likely to initiate an attack when the flower mantids stretched their forelegs (*χ*^2^ = 9.57, *p* = 0.002; figure 1*d*). These results indicate that foreleg stretching can significantly delay and reduce predator attacks, likely by intimidating the predators or confusing predatory mantids regarding the target location, which is akin to a phenomenon previously observed in butterflies [30–32].

Moreover, we found that mantids with stretched forelegs had an 82% survival rate after an attack. This suggests that the reduced attack rate alone cannot fully explain the higher survival rate in the foreleg-stretching group compared to the non-stretching group. Further analysis of the predator’s attack behaviour revealed that when flower mantids stretched their forelegs, predators more frequently directed their attacks towards the flower mantid’s forelegs (64%) than towards the body trunk (18%). In contrast, flower mantids that did not stretch their forelegs (*χ*^2^ = 7.61, *p* = 0.006) and those with disabled forelegs (*χ*^2^ = 16.74, *p* < 0.001) were nearly always attacked at the body trunk (figure 1*e*). Furthermore, attacks directed at the forelegs resulted in a significantly higher likelihood of escape compared to those directed at the body trunk (χ^2^ = 12.09, *p* = 0.001; figure 1*f*). Combined with the fact that foreleg stretching attracts the attention of predators (figure 1*b*), these results suggest that this behaviour can deflect predators’ attacks from the more vulnerable body trunk to the less vulnerable forelegs, thereby facilitating escape after the attack.

### (b) Foreleg stretching represents a behavioural co-option

**Figure 2.**
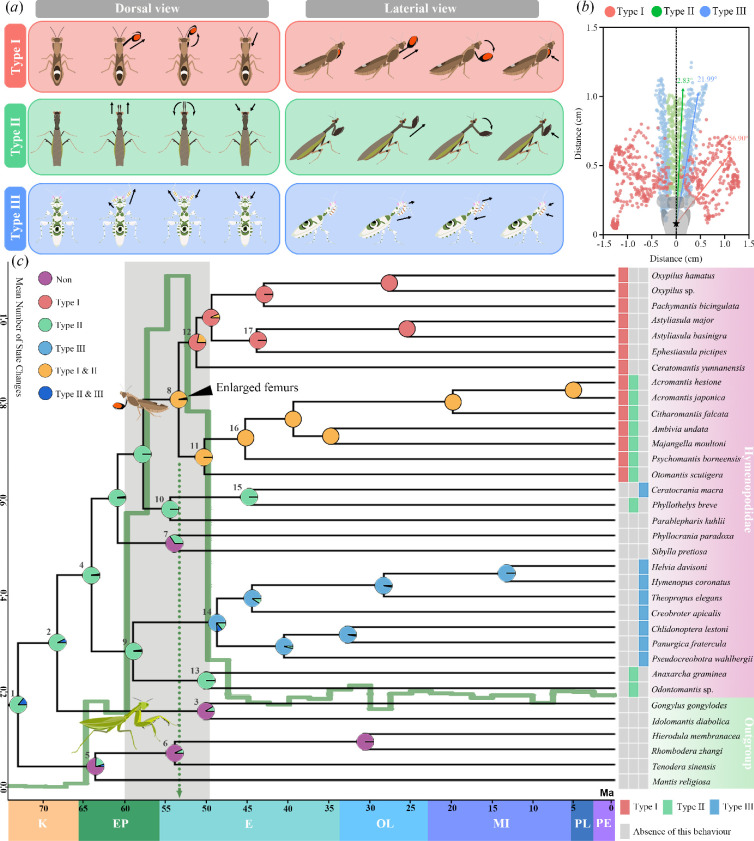
Diversity and evolution of foreleg-stretching behaviour in Hymenopodidae. (*a*) The three major types of foreleg-stretching behaviours. (*b*) The behaviour types were classified based on trajectories of the foreleg movement, which was recorded by tracking the apical points of foreleg femurs during the stretching movement. (*c*) Evolutionary history of the foreleg-stretching behaviour indicated by ancestral state reconstruction of the family Hymenopodidae. The numbers on the representative nodes indicate the divergence time, corresponding to electronic supplementary material, table S7. The states ‘Type I and III’ and ‘Type I and II and III’ are not labelled on the figure due to ignorable posterior probabilities. The green solid line in the background represents the stimulated mean number of state changes per 5 Ma. Coloured squares to the right of the phylogenetic tree represent the types of foreleg-stretching behaviour displayed by each species. The black triangle denotes the time when the most recent common ancestor of lineages exhibiting significant fore-femur expansion appeared (53.98 Ma, node 8), coinciding with the appearance of type I, slightly later than the beginning of the rapid evolution of stretching behaviours (approx. 60 Ma).

Comparative behavioural analysis across 25 genera of flower mantids revealed substantial interspecific variation in foreleg-stretching behaviours, which can be categorized based on the direction and extent of foreleg movement (figure 2*a*,*b*). Type I is characterized by the alternative extension of both forelegs to opposite sides of the body axis, with each leg extending at a large angle relative to the body’s central axis (approx. 56.90°). This mode is often accompanied by the shivering of the tibia and tarsus. Type II is the simplest of the three types, involving simultaneous forward stretching of both forelegs at a much smaller angle (approximately 2.83°) to the body’s central axis, making the inner surfaces of the forelegs concealed and resembling the predatory strike behaviour during hunting [13]. Type III involves the alternative extension and withdrawal of the forelegs in a circling motion, with the angle of stretching falling between that of type I and type II (approx. 21.99°). Notably, all three types can be spontaneously performed without external stimuli.

**Figure 3.**
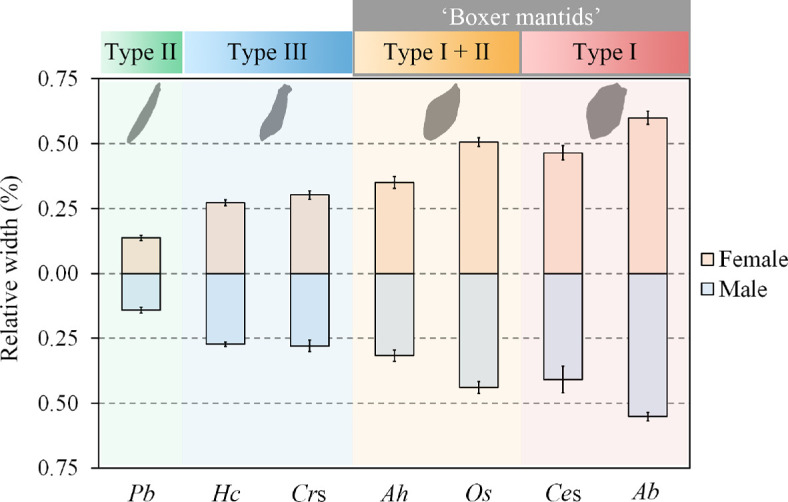
Correlation between foreleg-stretching behaviour and foreleg morphology. The level of expansion of foreleg femurs in lineages displaying different types of foreleg-stretching behaviours is measured as the ratio between the width and length of the foreleg femurs (mean ± s.d.). The abbreviations on the x-axis represent different species: *Pb*, *Phyllothelys breve; Hc*, *Hymenopus coronatus; Cr*s, *Creobroter* sp.; *Ah*, *Acromantis hesione; Os*, *Otomantis scutigera; Ce*s, *Ceratomantis* sp.; *Ab*, *Astyliasula basinigra*.

Ancestral state reconstruction for foreleg-stretching behaviour, based on the phylogeny of Hymenopodidae including all six subfamilies, revealed that the earliest form of the defensive behaviour is most likely type II (figure 2*c*; electronic supplementary material, figures S4 and S5), which is most similar to the prey-capturing stretch displayed by all mantids [13]. Comparative morphological analyses among flower mantid species displaying different types of foreleg-stretching behaviours revealed that at the early stage of the evolution of stretching behaviour, with the emergence of the type II behaviour, lineages exhibiting stretching behaviour showed almost no femur expansion. Greater femur expansion appeared following the evolution of the more derived stretching behaviour types, types I and III (figure 3; electronic supplementary material, table S6). This supports a major hypothesis in behavioural evolution and co-option, which predicts that changes in behavioural traits precede the evolution of associated morphological traits [12]. The flat, expanded surface of the foreleg femurs may enhance the conspicuousness of the marking display [49,50]. In addition, enlarged femurs may also impede predators’ abilities to capture and hold (electronic supplementary material, figure S6). These observations suggest that the functional shift of foreleg stretching may have driven the evolution of fore-femur morphology, making it better adapted to its novel function. Together, evidence from behavioural and morphological analyses supports our hypothesis that foreleg stretching serves as an anti-predator strategy co-opted from its ancestral predatory function.

### (c) The behavioural co-option of foreleg stretching might be driven by the emergence of visual ambush predators

**Figure 4.**
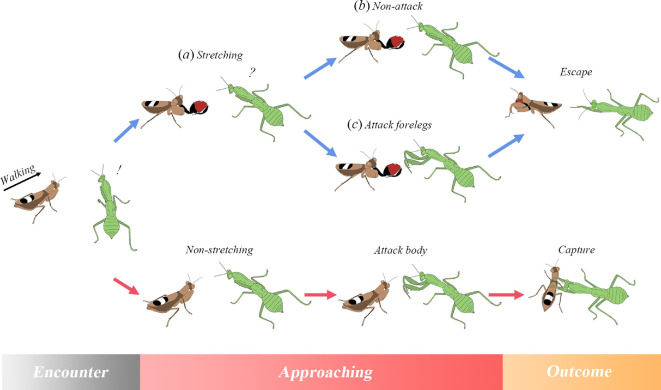
Inferred biological function of foreleg-stretching behaviour. Foreleg stretching can (*a*) slow down the predator approach, (*b*) restrain predator attack and (*c*) deflect a predator’s attack towards the foreleg, all of which may facilitate the escape of flower mantids from the predator.

Flower mantids are a group of small-sized mantids primarily found in subtropical and tropical regions. Both previous studies and our analysis suggest that flower mantids often cohabit with visual ambush predators such as larger mantid species (electronic supplementary material, figure S1), which are attracted to moving prey [21]. This may have generated a powerful driving force for this behavioural co-option to evolve. In line with the distribution data, our phylogenetic analysis dated the origin of the most complex stretching behaviours to 53.98 Ma (electronic supplementary material, table S7). Moreover, the evolutionary rate of the foreleg-stretching behaviour (as indicated by the simulated mean number of state changes of foreleg-stretching behaviours per 5 Myr) also peaked around the same timeframe (figure 2*c*; electronic supplementary material, figure S7). These events coincide with the timing of the radiation of the large mantids (Hierodulinae + Tenoderinae). Additionally, our behavioural observations suggest that foreleg-stretching behaviour is frequently exhibited even when flower mantids are actively moving, a state in which they are more likely to be detected by ambushing predators. In contrast, flower mantids barely stretch their forelegs (resting: 0.138 ± 0.153 times per minute, moving: 3.841 ± 1.590 times per minute; *t* = 6.13, *p* < 0.001; electronic supplementary material, figure S8) when at rest. This further suggests that foreleg stretching is primarily displayed to protect flower mantids against sudden, unexpected attacks, which are more likely to be elicited by ambushing mantids (figure 4). Therefore, our distributional, behavioural and phylogenetic data collectively support our hypothesis that the co-option of the stretching behaviour towards an anti-predator function is likely an evolutionary response to predation by visual ambush predators.

### (d) Does colour marking contribute to anti-predator defence?

Despite the above results, it remains unclear whether and to what extent the colour markings on the forelegs contribute to the anti-predator effects of foreleg stretching. In butterflies [30–32], tropical fishes [51,52] and some lizards and snakes [53–55], conspicuous markings play a key role in distracting and misleading bird and mammalian predators, which have strong colour vision and often select their prey based on colour cues [56,57]. However, in contrast to birds and mammals, praying mantids are not sensitive to long-wavelength colours [58] and are primarily attracted to moving objects. Therefore, if large mantids are the primary predators of flower mantids, the stretching movement of the forelegs may play a more important role than colour marking in protecting the mantids from these predators. Conversely, it is also possible that the colour markings are used for protection against potential bird or small mammalian predators [8,16,50]. Understanding the exact biological function of the colour markings and their contribution to anti-predator defence requires further behavioural studies and increased knowledge of the potential predators of flower mantids.

### (e) Behavioural co-option may be common across animals

By demonstrating behavioural co-option in an insect, our study expands current understanding of the taxonomic range of behavioural co-option and suggests that this phenomenon may be widespread across the animal kingdom. Given the challenges of cross-species comparative studies, relatively little attention has been paid to studying the behaviours of non-model organisms, particularly invertebrates. This lack of focus may partly explain the limited reports of behavioural co-option in the literature.

At the genetic level, ancient genes acquire novel functions through the modification of pre-existing genetic regulatory circuits established in ancestors [59]. Modularized cis- and trans-regulation of gene expression, through genetic switches, allows a single gene to be expressed at different times and locations, providing abundant opportunities for co-option [3–6]. Similarly, many behaviours can be expressed in multiple contexts, which may create ecological opportunities for co-option to occur. For example, some marking displays, originally intended for courting mates, can also be observed by unintended audiences such as predators [60]. If these unintended displays provide survival advantages under selective pressure, the behaviour may be co-opted for new functions.

Previous studies on behavioural co-option have primarily relied on field observations and often lack laboratory experiments that validate the functions of the behaviours under study [9–11]. Moreover, except for the well-documented case of the bowerbird, most studies do not provide information on the evolutionary history of the behaviour, which is critical evidence for the occurrence of co-option [12]. Our study offers a comprehensive empirical test of behavioural co-option by integrating manipulative behavioural experiments, multi-species comparisons and phylogenetic analyses. Phylogenetic methods have been extensively used to explore ecological factors driving lineage diversification [19,61,62], making them an ideal tool for testing behavioural co-option [12]. Our study emphasizes the advantages of a multidimensional approach to examining behavioural co-option and sets a precedent for future research in this field. Such an approach could facilitate the investigation of co-option at the behavioural level across diverse taxa, helping to uncover the patterns and ecological drivers of behavioural evolution.

## Data Availability

The newly obtained DNA sequence has been uploaded to the National Center for Biotechnology Information (see electronic supplementary material, table S2). Other data are accessible from the Dryad repository [63]. Supplementary material is available online [64].
